# Preparation and dye adsorption properties of activated carbon/clay/sodium alginate composite hydrogel membranes

**DOI:** 10.1039/d3ra07554k

**Published:** 2024-01-02

**Authors:** Nasim Ullah, Zarshad Ali, Amir Sada Khan, Bushra Adalat, Asma Nasrullah, Sher Bahadar Khan

**Affiliations:** a Department of Chemistry, Hazara University Mansehra 21120 Khyber Pakhtunkhwa Pakistan zarshad11@yahoo.com; b Department of Chemistry, University of Science and Technology Bannu 28100 Khyber Pakhtunkhwa Pakistan; c Department of Chemistry, Shaheed Benazir Bhutto Women University Peshawar 25000 Khyber Pakhtunkhwa Pakistan; d Chemistry Department, Faculty of Science, King Abdulaziz University P. O. Box 80203 Jeddah 21589 Saudi Arabia

## Abstract

A hydrogel membrane was prepared using activated carbon and sodium dodecyl sulphate modified montmorillonite clay incorporated into sodium alginate polymer. The activated carbon was prepared from a locally available susbine plant. The physiochemical characteristics of the synthesized hydrogel membrane were investigated using FTIR, SEM, EDX, and TGA techniques. The performance of the membrane was evaluated as an adsorbent by methyl red adsorption from water. The adsorption behavior of the hydrogel membrane was investigated under varying conditions of pH (2–10), membrane dose (0.0025–0.015 mg g^−1^), equilibrium adsorption time (30–360 minutes), solution temperature (25–45 °C) and dye concentration (100–500 mg L^−1^). The maximum adsorption capacity of the hydrogel membrane was 248.13 mg g^−1^. The kinetics of methyl red adsorption on hydrogel membrane best followed the pseudo-second order (PSO). The equilibrium adsorption results suggested that it obeyed the Freundlich isotherm very closely (*R*^2^ = 0.994). The thermodynamics of methyl red adsorption on the hydrogel membrane revealed that the adsorption was spontaneous (Δ*S*° = 16.15 kJ K^−1^ mol^−1^), favorable (Δ*G*° = −3.51 kJ mol^−1^), and endothermic (Δ*H*° = −1.48 kJ mol^−1^) in nature. These investigations suggested that the fabricated hydrogel membrane could be suitably used for methyl red adsorption from the solution.

## Abstract 2

A hydrogel membrane was prepared using activated carbon and sodium dodecyl sulphate modified montmorillonite clay incorporated into sodium alginate polymer.

## Graphical abstract

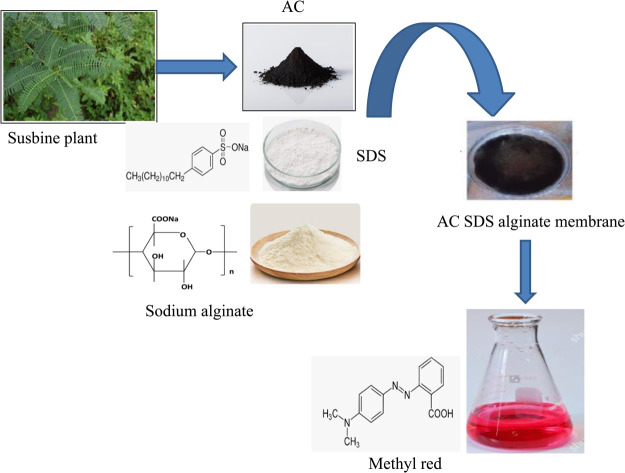

## 1. Introduction

A wide range of compounds can be found in wastewater from industries that cause environmental pollution and have toxic effects on human beings and animals if discharged without any treatment into water streams.^1^ All dyes were synthesized from chemical compounds, containing a particular color. They are chemically bonded to a substrate molecules, such as paper, fur, and fibers, to produce a beautiful color. They are often used in products, such as foods, beverages, cosmetics, medicines, and special care items, and in a variety of industrial sectors, such as paper, plastics, printing, textiles, and leather.^2^ Dyes are usually classified into anionic, cationic, and nonionic dyes. This classification is on the basis of the charge of the chromophore group when dissolved in the aqueous solution.^3^ Over 100 000 dyes are reportedly utilized in the printing and dyeing industries. Therefore, a significant amount of industrial effluents containing colors are released into the hydrosphere.^4^ The textile, paint, paper, and leather industries generate colored pollutants, which are a substantial source of water pollution. Dyes are highly visible even at very low concentrations (below 1 mg L^−1^) and affect phytoplankton photosynthesis.

Synthetic colorants in effluents are typically hard to get rid of because of their resistance and inability to degrade. They are thought to be mutagenic for living things in aerobic environments.^5^ At present, researchers are using various approaches to remove the dyes from wastewater.^6^ Usually, these methods include flocculation,^7^ membrane filtration,^8^ advance oxidation,^9^ ozonation and photocatalytic,^10^ biological treatment,^11^ and adsorption.^12^ These traditional methods have inherent flaws, such as complicated and ineffective technologies, which make it important to look for efficient and simple dye wastewater treatment methods.^13^

A bio-organic material called biomass originates from living organisms^14^ because it is a plentiful and renewable resource and it can be utilized as a substitute for raw material for the production of activated carbon.^15^ Pyrolysis is a thermo-chemical conversion process that uses heat to turn biomass into gas, liquid, and solid (activated carbon).^16^ An increase in the adsorption capacity can be achieved by optimizing the pyrolysis parameters, such as reaction temperature^17^ pressure^18^ heating rate,^19^ and residence time^20^ may alter the surface of a porous material and increase its surface area, all of which can improve the adsorption capacity.^21^ Several research projects are being undertaken to produce activated carbon from different biomass sources.^22^ However, in practice, it is challenging to replenish or regenerate activated carbon after usage, especially when it is in powder form. When activated carbon is added to water, it can eventually break down and produce additional pollutants. Therefore, including activated carbon in a suitable matrix might provide a substance with an excellent adsorption capacity and relatively easy post-use recovery.^23^ Methyl red is a water-soluble anionic dye and is regularly employed by the textile industry and other sectors. Therefore, in this research, a novel hydrogel membrane was prepared using boric acid (H_3_BO_3_) as an activating agent for the preparation of activated carbon. Then, prepared activated carbon and montmorillonite clay were incorporated into sodium alginate to from a hydrogel membrane using de-ionized water as a solvent and CaCl_2_ as a cross linking agent. FTIR, SEM, and TGA analyses were utilized to characterize the hydrogel membrane. The hydrogel was further used for the elimination of methyl red from water.

## 2. Materials and methods

Montmorillonite clay was sourced from the river Kasho location of Bannu, Pakhtunkhwa, Pakistan. Sodium hydroxide (NaOH 99%) was obtained from Merck Germany. While methyl red (C_15_H_15_N_3_O_2_) and hydrogen chloride (HCl 37%) were received from Sigma-Aldrich. Whereas, calcium chloride and sodium alginate (*M*_w_ 120 000–190 000 g mol^−1^) were also received from Sigma-Aldrich. All the chemical reagents were used without further purification.

### 2.1. Preparation of activated carbon (AC)

Susbine plant (SP) was used as the initial source for the preparation of activated carbon (AC). SP was broken down into tiny pieces. They were properly cleaned by repeatedly washing them in distilled water after being sliced into little pieces. These pieces were then dried for 15 hours at 110 °C in an oven before being ground into SP powder. Boric acid and SP powder were combined in a 1 : 3 (1 : 3 g g^−1^, SP : H_3_BO_3_) ratio. About 25 mL of distilled water was added to the combination of SP powder and boric acid (H_3_BO_3_). The mixture was stirred for two hours while it was at room temperature. After that, the mixture was heated for two hours at 500 °C in a muffle furnace. The sample was crushed when it was taken out of the furnace. To attain a neutral pH, the resulting sample was washed three times with 3 M HCl solution and several times with warm deionized water before baking it in an oven at 105 °C for 12 hours.

### 2.2. Modification of activated carbon with sodium dodecyl sulphate (MAC)

Sodium dodecyl sulphate (SDS) was used to modify the activated carbon. 30 mL of de-ionized water was combined with 0.5 g of activated carbon, and the mixture was stirred for an hour. The surface of activated carbon was then modified by continually adding 0.5 g of SDS and stirring for 2 hours. The resulting solution was then centrifuged and washed with de-ionized water to eliminate excess SDS and was kept for future use with modified activated carbon (MAC).

### 2.3. Preparation of MAC@montmorillonite clay and Ca-alginate hydrogel membrane

To form a hydrogel membrane, 0.5 g sodium alginate was slowly added to 50 mL of de-ionized water and stirred constantly for 4.5 hours. Afterward, 0.5 montmorillonite clay and 0.5 g of MAC were added continuously and stirred for 6 hours. Then, the hydrogel was sonicated continuously for 8 hours to completely homogenize the hydrogel. The homogenized hydrogel was added to 4% CaCl_2_ solution and stirred for 12 hours, then cast into a Petri dish to form a hydrogel membrane and washed with de-ionized water repeatedly to remove excess CaCl_2_ from the hydrogel membrane.

### 2.4. Characterization of the hydrogel membrane

The hydrogel membrane was characterized using different instrumental techniques. FTIR (PerkinElmer) analysis was performed in the range from 500–4000 cm^−1^ to determine various function groups present in the hydrogel membrane. The weight loss of the hydrogel membrane was identified through TGA (model, PerkinElmer STA 6000) in the presence of a nitrogen environment at 10 °C per minute. SEM (TESCAN VEGA, LUM analytical model) was used to analyse the surface morphology of the hydrogel membrane. The presence of key elements was determined using EDX (INCAx-act, Oxford) analysis attached to the SEM.

### 2.5. Adsorption studies of methyl red on the hydrogel membrane

The adsorption of methyl red was studied using batch adsorption tests. Methyl red stock solution (1000 ppm) was prepared in de-ionized water and diluted to prepare the required solution of methyl red. The adsorption of methyl red was investigated using different parameters such as methyl red concentrations, time, membrane dose, pH, and temperature, respectively. The amount of the hydrogel membrane was kept constant throughout these studies except in the membrane dose studies. 0.1 M solutions of HCl or NaOH were added dropwise to the methyl red solution to change the pH. Finally, the spectrophotometer (Shimadzu, 1800; Japan) was used to record the maximum adsorption capacity and percent adsorption of methyl red by using eqn (1) and (2), respectively.1
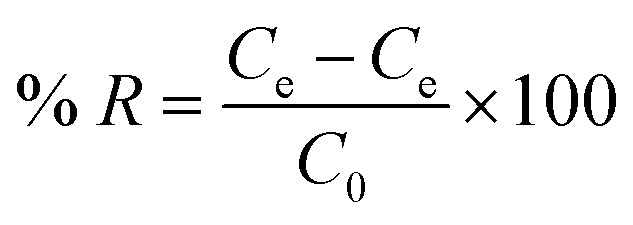
2
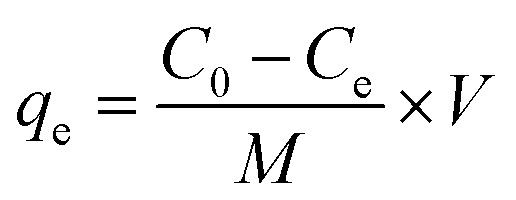


In the above equations, *C*_0_ is the initial concentration of methyl red, *C*_e_ is the equilibrium concentration and *M* is the mass of the hydrogel membrane, while *V* is the volume of methyl red solution.

## 3. Results and discussion

### 3.1. Characteristics of hydrogel membrane

**Fig. 1:**
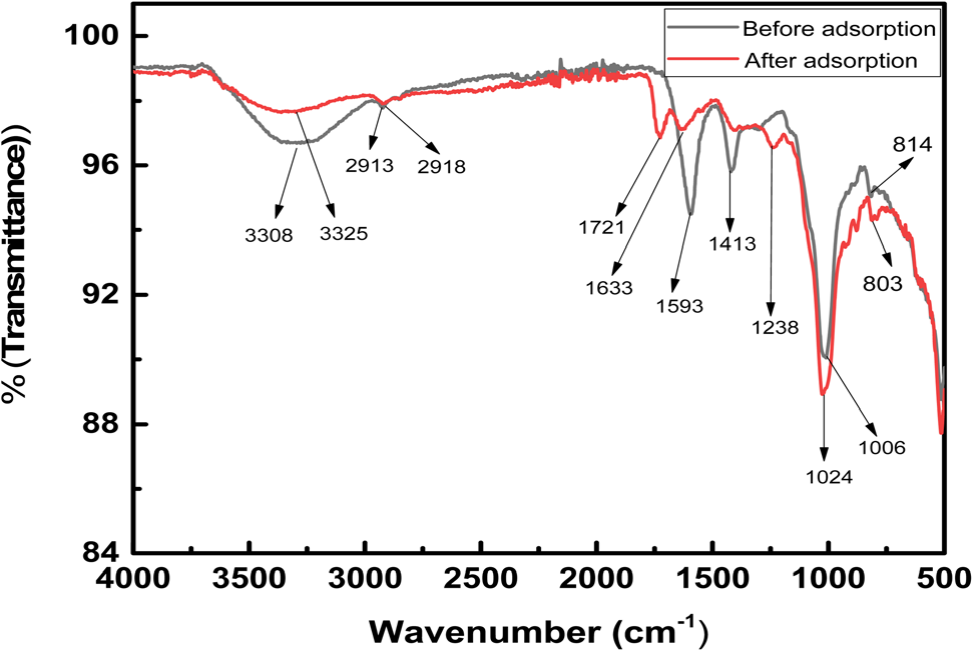
Spectra of pure hydrogel and after the adsorption of methyl red.

Fig. 1, shows the FTIR spectra of the hydrogel membrane before (a) and after (b) the removal of methyl red from solution and was recorded from 500–400 cm^−1^ to determine the function groups. The band at 1593 cm^−1^ was assigned to the aromatic ring vibration of the alkenes group (C=C).^24^ Peaks at 2913 and 2918 cm^−1^ were present before and after adsorption and they were caused by the C–H bond stretching on the surface of the hydrogel membrane.^25^ The presence of (O–H) stretching vibration peak was observed at 3308 cm^−1^, which shifted to 3325 cm^−1^ after adsorption. This shifting shows that methyl red was adsorbed successfully on the surface of hydrogel membrane.^26^ After adsorption of methyl red, the band at 1721 cm^−1^ corresponded to the presence of C=O of ester carbonyl vibration.^27^ The bands appearing at 1006 and 1024 cm^−1^ corresponded to the presence of the anti-symmetric vibration of the glycosidic linkage of (C–O–C) of the hydrogel membrane before and after adsorption.^28^ The existence of the band at 805 and 796 cm^−1^ is attributed to Si–O–Si and Si–O–Al in the amorphous hydrogel membrane, respectively.^27^ After the adsorption of methyl red, some new bands appeared, which signified that methyl red was successfully adsorbed on the hydrogel membrane.

**Fig. 2:**
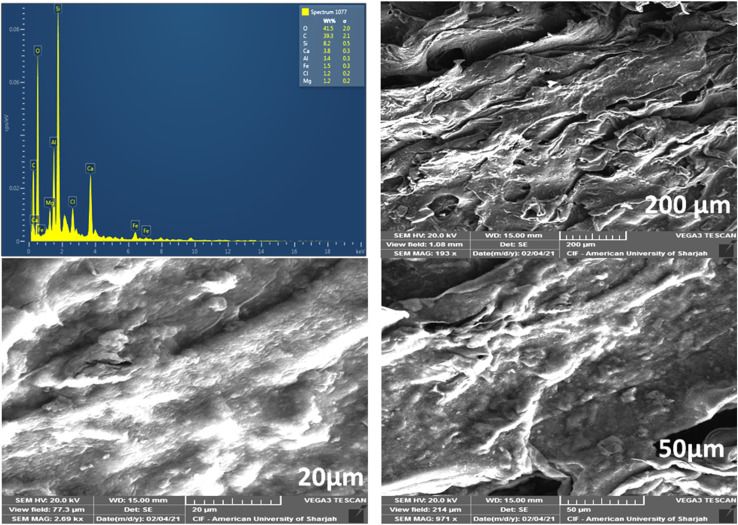
SEM and EDX pictures of the hydrogel membrane.

Fig. 2 shows the SEM images of the hydrogel membrane. The surface images of the hydrogel membrane showed that it had irregular, homogeneous, and rough surface. Besides this, it also has a large porous structure with cracks. These pores and cracks are responsible for the adsorption of methyl red from water.^29^ On the other hand, the EDX image of the hydrogel membrane revealed that different elements such as carbon, oxygen, iron, and magnesium were present in the hydrogel. The presence of these elements also helps in the removal of methyl red from water.^30^

**Fig. 3:**
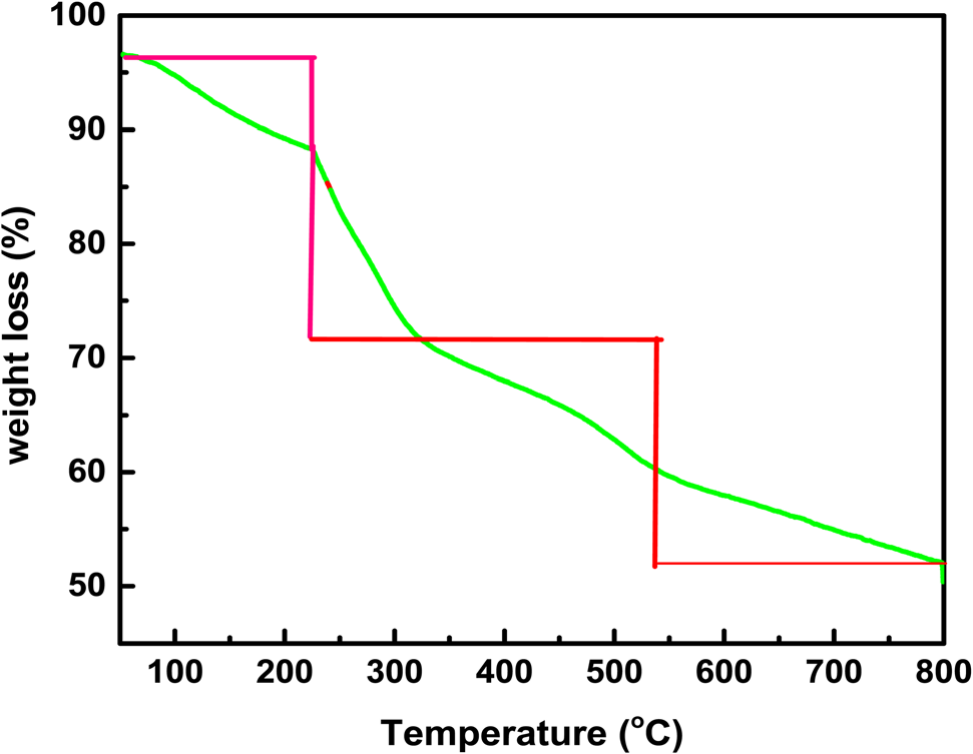
TGA spectra of the hydrogel membrane.

The TGA plot of the hydrogel membrane is depicted in Fig. 3. This plot shows that weight loss of the hydrogel membrane occurred in four steps. In the temperature range of 50–230 °C, approximately 10% weight loss was observed for the hydrogel membrane. This may be due to the loss of moisture from the hydrogel membrane and methyl red as well as the dehydration of montmorillonite clay. In the second step, from 230–350 °C, approximately 18% weight loss occurred. This was due to the decarboxylation and dihydroxylation of the hydrogel membrane. In the third step, from (350 to 560) °C, approximately 10% of the weight loss occurred from the hydrogel membrane. This loss was due to the volatilization and disintegration of constituents of the carbon chain in the hydrogel membrane. In the final step, from 560–800 °C, about 8% of weight loss occurred due to the removal of organic compounds from the hydrogel membrane such as lignin.^31^

### 3.2. Adsorption of methyl red on the hydrogel membrane

#### 3.2.1. pH effect

**Fig. 4:**
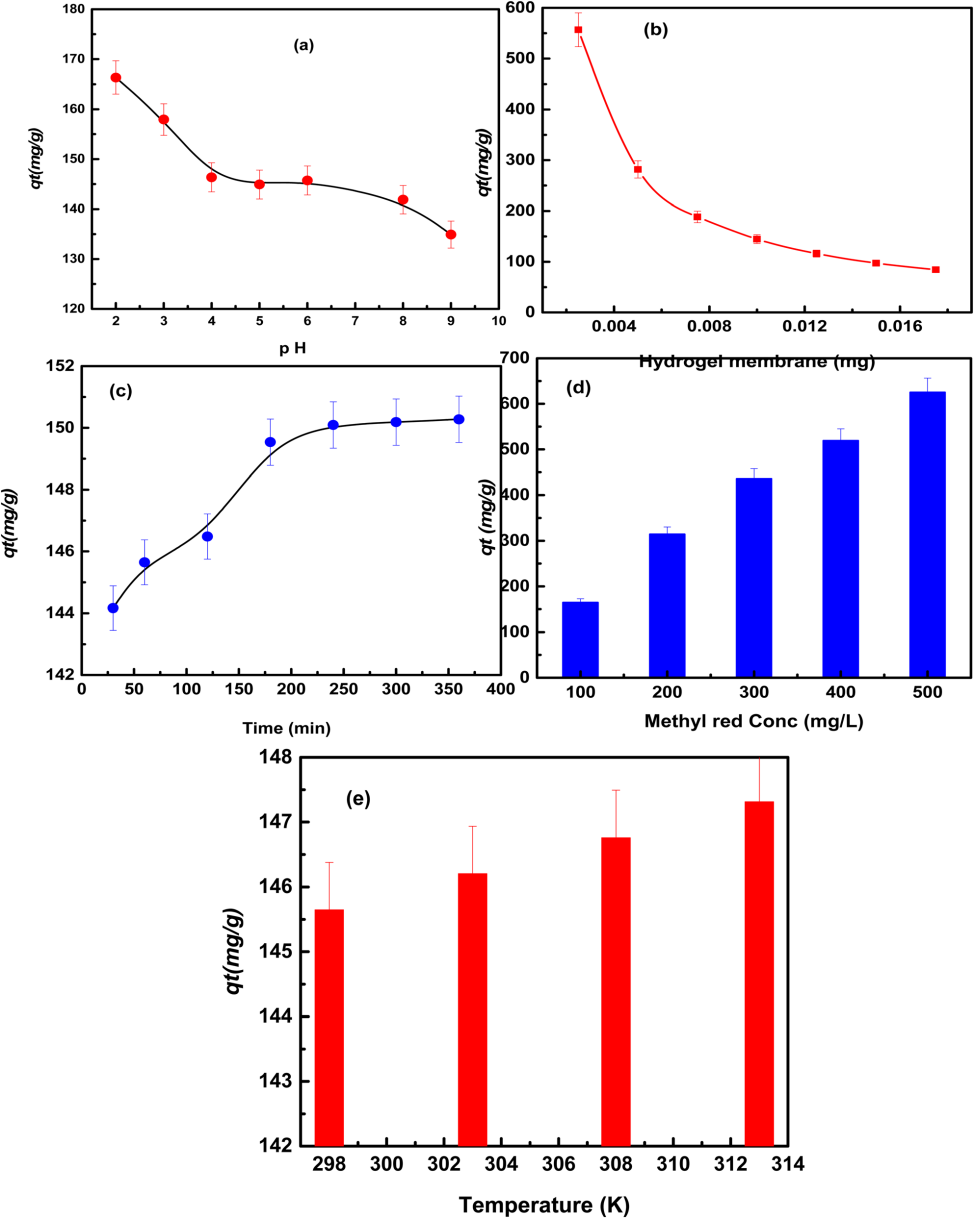
The effect of pH (a), hydrogel membrane (b), time (c), methyl red conc. (d), and temperature (e) on methyl red adsorption on the hydrogel membrane.

The pH for the methyl red adsorption on the hydrogel membrane was studied from pH 2–10 using 100 mg L^−1^ of methyl red, adsorbent dose of 0.01 g, at room temperature. The obtained results are shown in Fig. 4(a). These results showed that the percent adsorption of methyl red increased to 166.33 mg g^−1^ and then decreased slowly to 134.91 mg g^−1^ as the pH of the solution increased. Because of the protonation, the hydrogel membrane surface was positively charged at low pH. However, the surface was deprotonated resulting in the negative charge as the pH was increased. The enhanced adsorption of the dye at pH 2 may be due to the electrostatic interaction between the negatively charged dye molecules and the positively charged adsorbent surface. At high pH, dye elimination was lowered possibly due to the electrostatic repulsion between the negatively charged dye molecules and the negatively charged adsorbent surface. In this study, the maximum removal was achieved in an acidic region at pH 2. Therefore it was chosen for further adsorption studies.^25^

#### 3.2.2. Membrane adsorbent dose effect

In order to study the effect of methyl red adsorption, various amounts (0.0025–0.020 g) of the hydrogel membrane were tested at pH 2 using methyl red concentration of 100 mg L^−1^ at 25 °C as shown in Fig. 4(b). These results indicated that the percent removal increased very quickly when the amount of the hydrogel membrane was increased. This means that at the beginning of the reaction more vacant active sites were available on the surface of the hydrogel membrane. Due to the availability of more vacant sites, methyl red molecules were easily attached to the hydrogel membrane. Therefore, there was an increased percent removal of the dye from 55.66 to 59.07 mg g^−1^.^32^ Whereas, the adsorbed quantity of methyl red decreased from 556.66 to 84.39 mg g^−1^. This was due to the presence of the unoccupied sites of the hydrogel membrane during the adsorption of methyl red molecules, the adsorption efficiency declined as the adsorption dosage was increased.^33^

#### 3.2.3. Effect of time

100 mg L^−1^ of methyl red in a pH 2 solution was used to test the impact of the adsorption time on the removal of methyl red using 0.01 g amount of the hydrogel membrane, while the time of shaking was varied from (30–360) minutes at room temperature (25 °C) and the outcomes are depicted in Fig. 4(c). The removal capacity of methyl red was increased from (144.16 to 150.27 mg g^−1^) as the time was increased continuously up to 360 minutes and after that no change was observed in the adsorption. This means that a large number of active sites were present in the hydrogel membrane surface initially, which attracted methyl red molecules, and hence the adsorption efficiency increased. Within 360 minutes, all the active sites in the hydrogel membrane were occupied by methyl red molecules and hence more methyl red molecules could not be accommodated. Therefore, equilibrium was achieved after 360 minutes. Equbal Ahmad Khan *et al.* (2018), have also reported similar trends for methyl red adsorption on a biosorbent.^25^

#### 3.2.4. Effects of methyl red concentration

The effect of methyl red concentrations (100–500 mg L^−1^) was studied to determine the adsorption efficiency of the hydrogel membrane. The time of shaking was varied over 24 hours at pH 2, and 0.01 g of the hydrogel membrane was used at 25 °C. Fig. 4(d) shows that the removal efficiency of the hydrogel membrane was increased from 154.44 to 864.72 mg g^−1^ as the concentrations of methyl red were increased. The adsorption efficiency of methyl red increases with increasing concentrations because more molecules come in contact with the surface as the methyl red concentration increases. Strong driving forces and the quick transfer of methyl red molecules from the aqueous to the solid phases are facilitated at enhanced methyl red concentrations.^34^

#### 3.2.5. Temperature studies

**Fig. 5:**
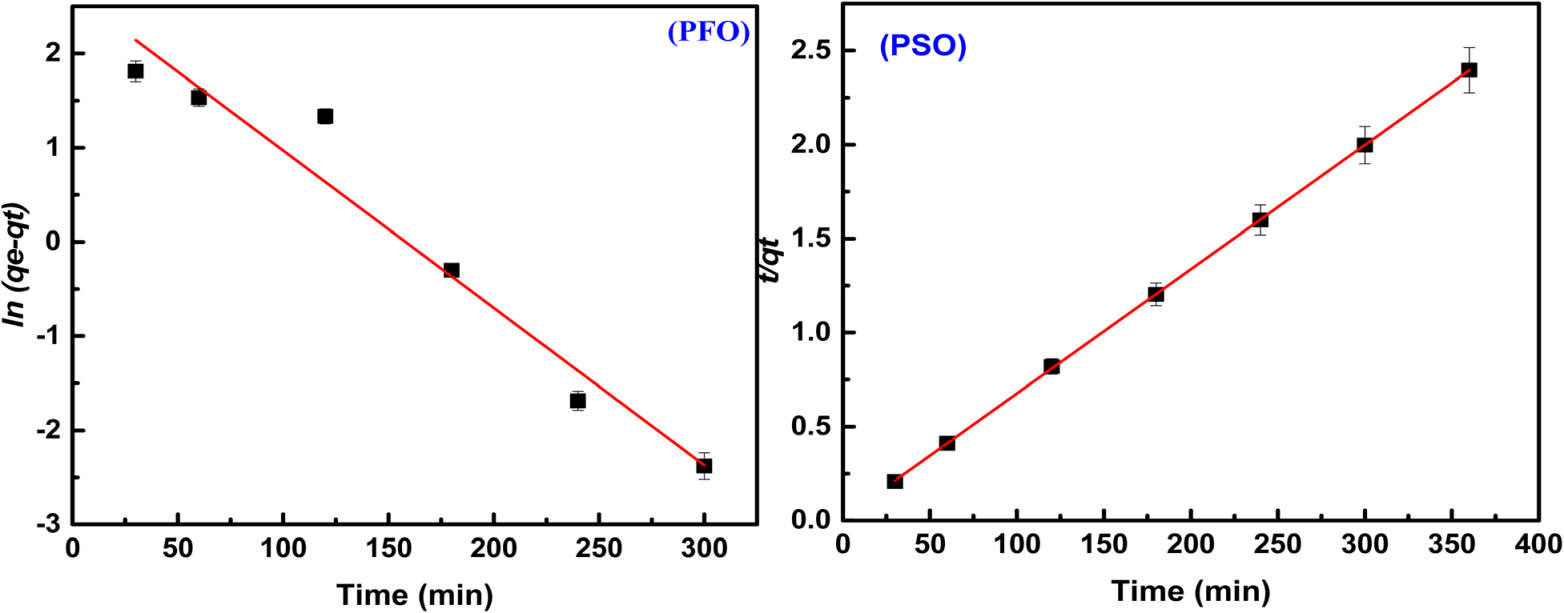
Pseudo first and second order kinetic models for methyl red adsorption on the hydrogel membrane.

The effect of temperature on the adsorption of methyl red on the hydrogel membrane was studied at 298 to 318 K, using 100 mg L^−1^ of methyl red at pH 2 and an adsorbent dose of 0.01 g, as illustrated in Fig. 5(e). It was observed that, with increasing temperature (25–45 °C), the elimination of methyl red from the hydrogel membrane increased from 225.5 to 232.5 mg g^−1^. This increase in the adsorption capability with the increase in the temperature was attributed to the increase in the kinetic energy of the methyl red molecules, which accelerates their approach to the hydrogel membrane surface. Furthermore, high temperatures generate membrane swelling and enhance the surface area of the hydrogel membrane.^35^ Another possible reason is that, with an increase in temperature, a number of active sites may be produced in the hydrogel membrane which increases methyl red adsorption. The results obtained indicated that methyl red adsorption on the hydrogel membrane at high temperatures was favorable, spontaneous, and endothermic in nature.^36^

#### 3.2.6. Kinetics study

The rate of adsorption mechanism of methyl red on the hydrogel membrane was investigated using pseudo-first order (PFO) and second order (PSO) kinetic reactions. Initially, PFO and PSO kinetic models were proposed by Lagergren.^37^ The linear forms of these two kinetic models are generally expressed in the following eqn (3) and (4).

3 ln(*q*_e_ − *q*_*t*_) = ln *q*_e_ − *k*_1_*t*

4
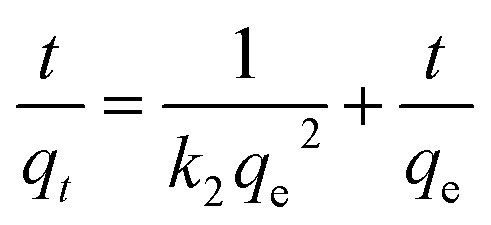


**Fig. 6:**
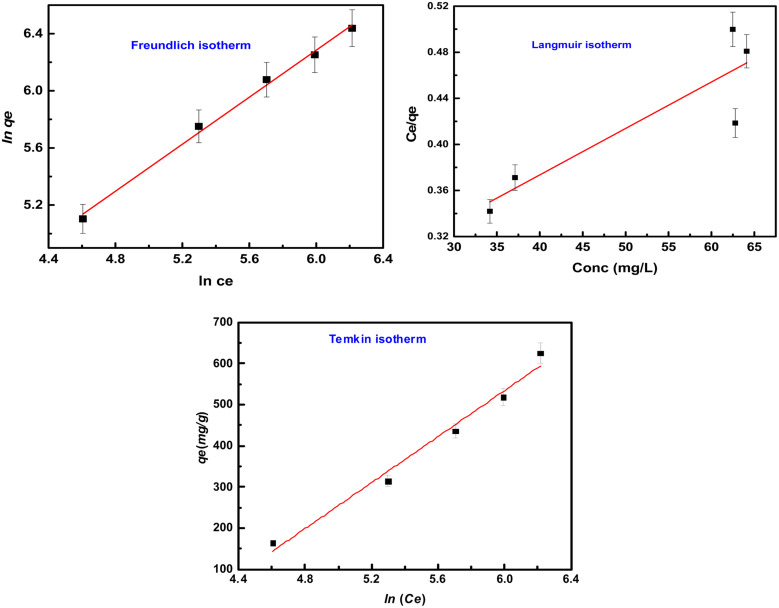
Langmuir, Freundlich and Temkin isotherm plots for methyl red adsorption on the hydrogel membrane.

In the above equations, *q*_e_ and *q*_*t*_ (mg g^−1^) represent the adsorbed quantity of methyl red on the hydrogel membrane at equilibrium and with respect to time *t*, respectively. While *k*_1_ (min^−1^) and *k*_2_ (g mg^−1^ min^−1^) represent rate constants for pseudo 1^st^ and 2^nd^ orders, respectively. The intercept and slope of *t*/*q*_*t*_*vs.* time were used to calculate the values of *q*_e_ and *k*_2_. Fig. 6 represented 1^st^ and 2^nd^ order kinetic equilibrium fitting results for methyl red adsorption. The parameter values calculated from PFO and PSO as well as *R*^2^ values are depicted in Table 1. The experimental value of the hydrogel membrane (150.13 mg g^−1^) was approximately equal to the calculated value (151.13 mg g^−1^). Whereas, Table 1 shows that *R*^2^ (0.955) of the pseudo 1^st^ order kinetic is less than that of the pseudo 2^nd^ order (0.999) reaction. This higher value of *R*^2^ (0.999) shows that PSO for methyl red adsorption on the hydrogel membrane is better fitted than the PFO reaction. Similar trends for methyl red adsorption on other surfaces are also reported.^38^

**Table 1:** Pseudo first and second order parameters for methyl red adsorption

Kinetic models	Parameters	Numerical values
Pseudo first order	*k*_1_ (min^−1^)	6.0 × 10^−3^
	*q*_cal_ (mg g^−1^)	13.80
	*R* ^2^	0.955
Pseudo second order	*k*_2_ (mg g^−1^ min^−1^)	2.0 × 10^−3^
	*q*_cal_ (mg g^−1^)	151.74
	*R* ^2^	0.999

#### 3.2.7. Isotherm studies for methyl red adsorption

Different isotherms such as Langmuir, Freundlich, and Temkin were applied to the experimental data to find out the mechanism of the interaction between methyl red and hydrogel membrane as well as maximum adsorption in the aqueous solutions. Langmuir, Freundlich, and Temkin models in linear forms are generally expressed as eqn (5–7) respectively.5
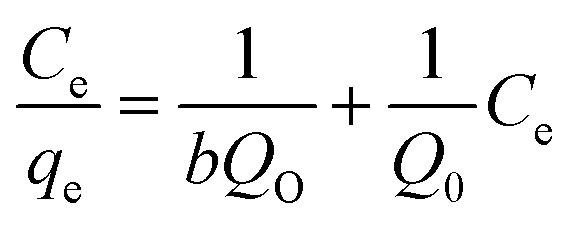
6
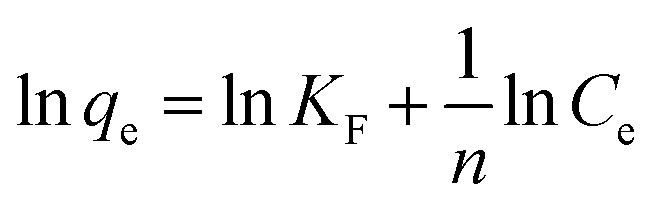

**Fig. 7:**
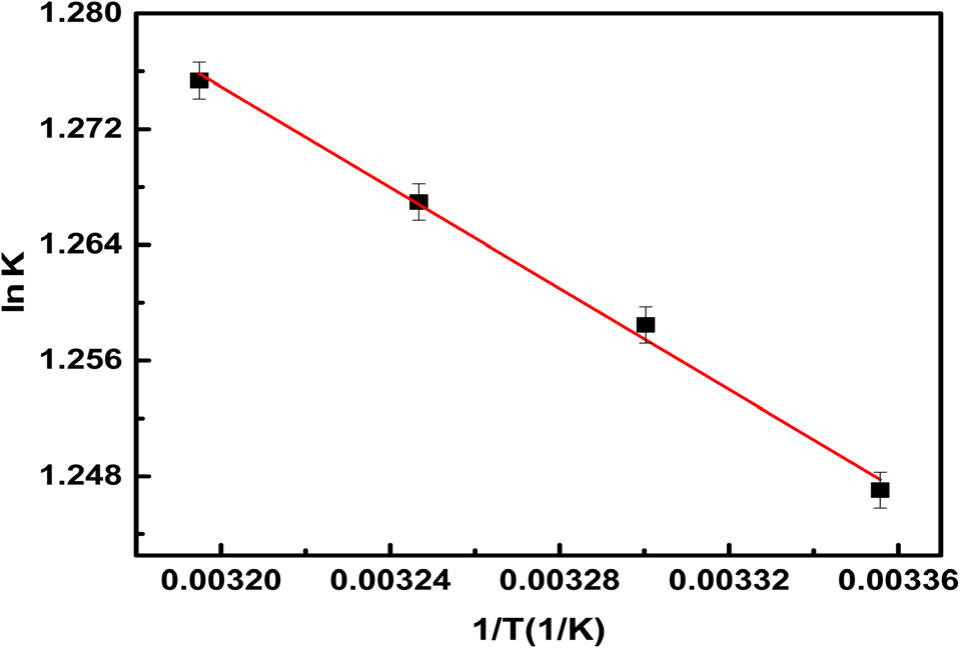
Van't Hoff plot for methyl red adsorption on the hydrogel membrane.

7 *q*_e_ = *B* ln *K*_T_ + *B* ln *C*_e_

where *q*_e_ is adsorption capacity at equilibrium and *C*_e_ is the equilibrium concentration of methyl red. Freundlich constant is donated by *K*_F_, and the adsorption intensity is represented by *n*. While *K*_T_ represents the adsorption equilibrium constant, *B* = *RT*/*b* is related to the enthalpy of adsorption^39^ and *b* is the adsorption heat for Temkin constant (J mol^−1^) as well as *R* is gas constant, and is equal to 8.314 J K^−1^ mol^−1^ and *k* is the absolute temperature. The graphical representation of these three isotherms for the removal of methyl red on the hydrogel membrane is shown in Fig. 7. The obtained isotherm constants values are depicted in Table 2. Freundlich isotherms were best fitted to the adsorption data and the obtained regression coefficient (*R*^2^ = 0.994) was higher than that of Langmuir isotherm (*R*^2^ = 0.799) and Temkin isotherm (0.975). The higher *R*^2^ value indicated that methyl red adsorption on the hydrogel membranes follows the Freundlich isotherm and that methyl red adsorption occurs as a monolayer on the hydrogel membrane surface.

**Table 2:** Langmuir and Freundlich constants for methyl red removal

Isotherm	Parameters	Numerical values
Freundlich	*n*	1.21
	*K*_F_	3.84
	*R* ^2^	0.994
Langmuir	*q*_e_ (mg g^−1^)	248.13
	*K*_L_ (mg L^−1^)	0.01
	*R* ^2^	0.799
Temkin	*B*	279.21
	*K*_T_	8.17
	*R* ^2^	0.975

#### 3.2.8. Adsorption thermodynamics

The thermodynamic feasibility and spontaneity of methylene red adsorption on the hydrogel membrane, such as the change in Gibbs free energy (Δ*G*°) and entropy (Δ*S*°) as well as the change in enthalpy (Δ*H*°) were also calculated using eqn (7) and (8).8
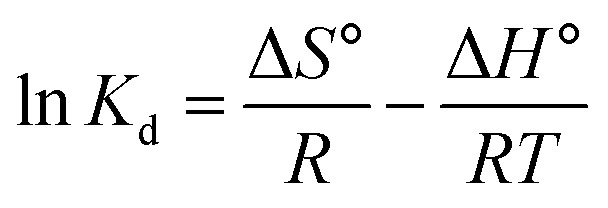

9 Δ*G*° = Δ*H*° − Δ*S*°*T*

In the above equations, the distribution constant for the adsorption of methyl red is represented by *K*_d_ (L mg^−1^), *R* represents the gas constant, and *T* is the absolute temperature (K). Table 3 shows, the obtained results of Gibbs free energy (Δ*G*°), Δ*H*°, and Δ*S*° for methyl red adsorption. The results obtained from Gibbs free energy (Δ*G*°), as shown in Table 3 display that when the temperature increased the negative values of Gibbs free energy (Δ*G*°) also increased. This negative value of (Δ*G*°) results reveals that the removal process from the water solution on the hydrogel membrane was spontaneous in nature and adsorption will be favorable thermodynamically. While the calculated value of Δ*H*° was (−1.48 kJ mol^−1^). This showed that the removal processes were exothermic in nature. As a result, at much higher temperatures more methyl red molecules will be released from the membrane surface and will result in desorption. The positive value of Δ*S*° shows increased randomness at the solid-solute surface and that the entropy gained by the water molecules is smaller than the entropy lost by methyl red molecules.^40^

**Table 3:** Thermodynamic constants for methyl red removal

Temperature (K)	Δ*G*° (kJ mol^−1^)	Δ*H*° (kJ mol^−1^)	Δ*S*° (kJ mol^−1^ K^−1^)
298	−3.27	−1.53	16.15
303	−3.36		
308	−3.43		
313	−3.51		

#### 3.2.9. Regeneration and reusability study

**Fig. 8:**
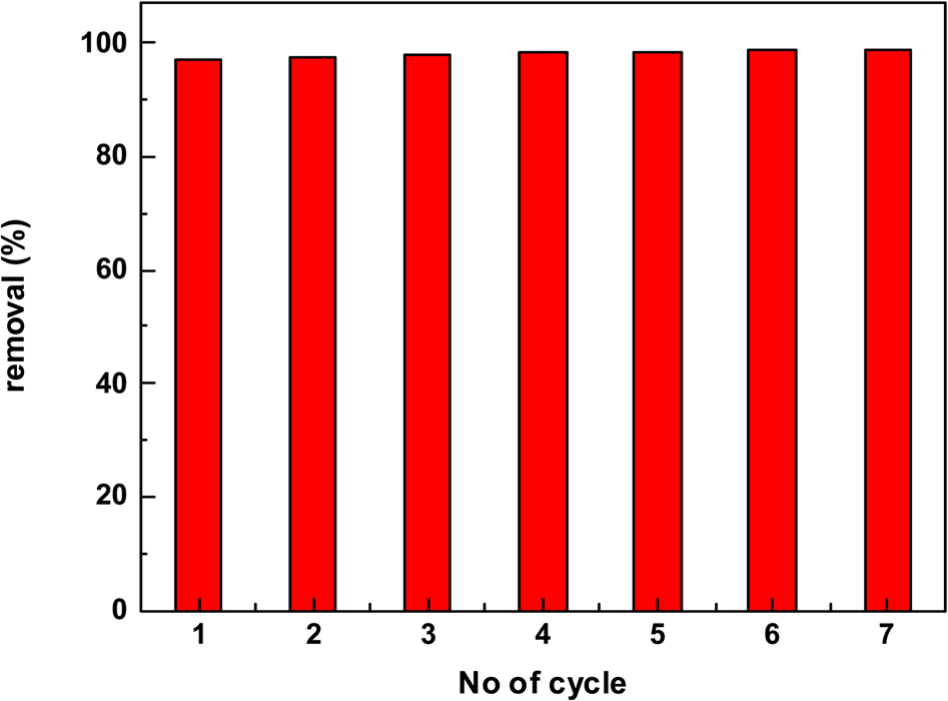
Regeneration and reusability of the hydrogel membrane.

In order to explore the prepared hydrogel membrane at the pilot scale, regeneration, and reusability are essential parameters. The result obtained from Fig. 8, shows that after several sorption desorption cycles the adsorption capacity of the membrane did not alter much and remained stable. The adsorption capacity was slightly increased from 96.95 to 98.96 mg g^−1^ after seven cycles. This means that the interior of the recycled hydrogel membrane may have been altered, which could clarify the increase in the absorption capacity of the hydrogel membrane. As a consequence of the reusability and regeneration results, the synthesized membrane retained high MB removal effectiveness after multiple successive adsorption cycles, offering the most effective response for broad industrial applications.

#### 3.2.10. Comparison of the synthesized hydrogel membrane with other membranes/adsorbent

The maximum capacity for the methylene blue adsorption on the hydrogel membrane prepared in this work was 248.14 mg g^−1^. This outcome was compared to several adsorbents produced by other researchers, as demonstrated in Table 4. When compared to other adsorbents such as clay–alginate surfactant, activated organo-bentonite, AC/alginate beads, and chitosan/montmorillonite (CTS/MMT), the synthesized hydrogel membrane demonstrated a greater capacity for adsorption. Some of the reported membranes show a decrease in the adsorption capacity while some of the other membranes may rupture after adsorption.^41^ Even though, the absorption capacity of the synthesized hydrogel membrane may be slightly lower than that of a few other adsorbents reported in the literature, other benefits of the reported adsorbents such as local availability, abundance as the precursor, nontoxicity, and low cost might compensate for a few disadvantages.

**Table 4:** Comparison of maximum adsorption capacity the synthesized membrane with other adsorbents

Adsorbents	Dyes	Adsorption capacity *A* (mg g^−1^)	References
Carbon clay/alginate membrane	MR	248.14	This work
Activate clay–chitosan	MO	330	42
CTS/MMT	MO	70	43
AC/alginate beads	MB	230	33
Clay	MB	270	44
Clay–alginate surfactant	MB	109.9	45
Activated organo-bentonite	MO	40.42	46

## 4. Conclusions

In this research work, activated carbon was prepared from a locally available susbine plant using H_3_BO_3_ as an activating agent. After preparation, the activated carbon was incorporated into sodium dodecyl sulphate MMT clay and sodium alginate to form the hydrogel membrane. The hydrogel membrane was further used for the adsorption of methyl red from the solution. Numerous adsorption factors, such as pH, dose, concentration, time, and temperature were investigated. The maximum adsorption capacity was 248.13 mg g^−1^. The removal of methyl red on the hydrogel membrane may be best fitted by a pseudo-second-order rate equation. While the equilibrium study showed that, it followed Freundlich isotherm. The Δ*G*° negative values of the results revealed that the adsorption process was spontaneous and favorable at high temperatures for methyl red adsorption onto the prepared hydrogel membrane. The calculated negative Δ*H*° value showed that the removal was an exothermic process in nature. While the Δ*S*° value indicated the increased randomness of solid-solute at the interface. In a nutshell, the fabricated hydrogel membrane was found to be an effective adsorbent for the decontamination of methyl red from water solutions.

## Conflicts of interest

The authors declare no conflict of interest.

## Supplementary Material
